# Geroscience Meets Medical and Social Science: Explaining Differential Aging Health 

**DOI:** 10.1093/geroni/igaf122.682

**Published:** 2025-12-31

**Authors:** Eileen Crimmins

**Affiliations:** University of Southern California, Los Angeles, California, United States

## Abstract

Geroscience has hypothesized that all age-related health outcomes are related to a set of age-related biological processes that combine to produce health deterioration with age. Medical science has been developing an increasingly wider array of risk factors for age-related health outcomes. Social science has long focused on differential aging; i.e. aging that occurs at different rates for population subgroups, those exposed to adverse social conditions, or health behaviors that put them at risk. Using insights from geroscience and medical science, I will provide greater clarity to how the social world gets under the skin to produce differential rates of aging through the mediation of biological processes. This will be done through the integration of biological measures at two levels – the molecular and cellular features included in the “Hallmarks of Aging” and risk factors identified at the biochemical or clinical level – into models assessing social differences in important health outcomes linked to age.

